# Retrospective analysis of a surgical service in a rural district hospital in the Eastern Cape

**DOI:** 10.4102/safp.v68i1.6226

**Published:** 2026-02-19

**Authors:** Jessica M. Westwood, Jocelyn Park-Ross, Rowan Duys

**Affiliations:** 1 Global Surgery Division, Department of Surgery, Faculty of Health Sciences, University of Cape Town, Cape Town, South Africa; 2 Madwaleni District Hospital, Elliotdale, South Africa; 3 Department of Anaesthesia and Perioperative Medicine, Faculty of Health Sciences, University of Cape Town, Cape Town, South Africa

**Keywords:** district hospitals, essential and emergency surgical care, surgical capacity, rural South Africa, training, surgical workforce, family physicians, surgical service delivery

## Abstract

**Background:**

District hospitals (DHs) are essential providers of surgical care in low- and middle-income countries. Despite recommendations to strengthen DH surgical services, data on South African DH surgical capacity remain limited. This study describes the volume, scope and workforce of surgical services at a rural Eastern Cape DH over 7 years.

**Methods:**

A retrospective audit of all surgical procedures (January 2016–December 2022) was conducted using theatre register data. Patient demographics, procedure type and surgical provider were extracted to analyse trends in surgical volume, scope and workforce.

**Results:**

A total of 2616 operations were performed, predominantly in females (97%), with a median age of 25 years. Statistical process control analysis showed a significant upward shift in the mean monthly surgical volume from 27 to 41 procedures. The surgical scope expanded from 14 different types of procedures in 2016 to 25 in 2022, covering obstetrics, gynaecology, general surgery, orthopaedics and urology. Caesarean sections accounted for 82% of procedures. Family medicine registrars and specialists performed the highest number of procedures per person.

**Conclusion:**

Surgical services expanded in both volume and scope, demonstrating the capacity of district-level facilities to meet essential surgical needs.

**Contribution:**

This study provides rare longitudinal data on rural South African DH surgical services, highlighting the critical role of decentralised family medicine training and senior staffing in supporting surgical expansion and strengthening district-level care.

## Introduction

One-third of the global burden of disease necessitates surgical, obstetric or anaesthetic care.^1^ However, in low- and middle-income countries (LMICs), 90% of people either lack access to this care or face catastrophic economic consequences to obtain it.^2^ To address this issue, the World Health Organization (WHO) and the Lancet Commission on Global Surgery (LCOGS) advocate for enhancing the surgical capacity of district hospitals (DHs).^2^ This cost-effective solution reduces the burden on higher-level facilities, improves patient accessibility and minimises delays.^3,4^

South Africa’s public sector health services are provided within a district health system, with the DH often being the first entry point for patients seeking surgical care. District hospitals constitute a heterogeneous category of healthcare institutions, exhibiting significant variations in staffing, infrastructure and capacity to deliver the package of surgical care suggested by global bodies and local regulators. Comprehensive data on the readiness of South African DHs to provide an essential surgical care package are lacking.^5^ Existing data highlight the impact of insufficient delivery of essential and emergency surgical, obstetric and anaesthetic care, resulting in avoidable morbidity and mortality.^6,7,8^

To improve access to emergency and essential surgical care (EESC), global organisations such as the World Bank and the WHO have recommended specific surgical procedures which should be offered at DHs.^3,9^ Several African countries are developing or have established National Surgical, Obstetric and Anaesthesia Plans.^10^ South Africa has sought to implement a DH service package outlining procedures suitable for DHs.^11^ Tools such as the bellwether procedures and the World Health Organization Surgery Assessment Tool (WHO SAT) have been designed to assess system readiness and outputs.^12,13^ Despite these efforts, examples of sustained improvements in surgical service delivery remain elusive. This may be because of the limited effectiveness of top-down directives compared to strategies that engage local healthcare providers through group problem-solving and training initiatives, drawing on the intrinsic motivation of local change agents with a desire to improve and expand DH surgical services.^14,15^

Our research aims to detail the surgical volume, scope and workforce at a rural DH in the Eastern Cape of South Africa. By examining changes in surgical service delivery over time, we seek to identify contextual factors that facilitate effective change. We hypothesise that improvements in the surgical volume and scope are achievable through a combination of relevant contextual factors and targeted local interventions, and that specific workforce cadres bear a disproportionate burden of these outputs.

## Research methods and design

### Clinical setting

This study was performed at Madwaleni District Hospital in South Africa’s Eastern Cape, which has features likely to be representative of other rural DHs. It is a medium-sized, government-funded DH with 180 inpatient beds, a casualty unit and a busy outpatient department. The hospital serves approximately 100 000 people spread over a vast geographical area, making access an often lengthy and expensive task.^16,17^ Additionally, this population suffers from high rates of unemployment, low literacy rates, poverty and lack of water, electricity and sanitation.^17^

The hospital provides a wide range of clinical services, including adult medicine and surgery (inclusive of orthopaedics and trauma), paediatrics, neonatology and obstetrics. A single team of clinicians (doctors and clinical associates) provides clinical care across all these domains. In March 2017, a decentralised family medicine training programme was launched at the hospital, offering registrars in family medicine a 4-year specialisation based at this facility under the supervision of an accredited university. The primary referral centre is an academic hospital located 2 h away by poor-quality roads, posing significant economic and logistical barriers to accessing higher levels of care. There are no other public or private healthcare facilities offering surgical care in the vicinity.

Surgical services at this facility are delivered across two primary locations. The main operating theatre is prioritised for major cases and obstetric emergencies (Caesarean sections [CSs]). To keep the main theatre available for emergencies, a dedicated ‘minor procedures room’ within the emergency unit functions as a sterile environment for other surgical procedures. Consequently, sterile cases such as tendon repairs, debridements and fracture washouts are frequently performed in this minor theatre.

### Instrument and justification

We conducted a retrospective audit of operating theatre activity at Madwaleni District Hospital from January 2016 to December 2022. This 7-year timeframe was selected to capture variations in surgical volume and scope of procedures over time.

Data for this review were sourced exclusively from the official operating theatre register. The inclusion criterion was all surgical cases performed within the operating theatre and recorded in this register during the study period. Surgical procedures performed in other hospital areas, such as the casualty department, were not captured in the theatre register and were therefore explicitly excluded from this analysis. For the purposes of this study, a ‘surgical procedure’ was operationally defined as any clinical intervention requiring the use of the main operating theatre environment and subsequently recorded in the theatre register.

Descriptive data captured by theatre nursing staff during each theatre case were extracted from the register. The variables collected included the procedure date, patient age, patient sex, cadre of the primary surgeon, cadre of the anaesthetist, start and finish times and the type of procedure. Data regarding surgical assistants were not routinely recorded in the theatre register and were therefore not included in the analysis. Clinician and patient data were de-identified. Any inconsistencies in the data were clarified by cross-referencing with clinical records and other service-level data sources. Data for each case were limited to the primary procedure listed in the register. In cases where multiple procedures were recorded (e.g. a CS and a bilateral tubal ligation [BTL]), only the primary procedure (the CS) was extracted for this analysis. Standalone procedures, such as a BTL performed as the primary operation, were recorded as such.

Supplementary data were obtained and verified by the internal human resources department and the District Health Information System. This included the annual medical staff complement and their professional cadre. Local census data from Statistics South Africa were used to estimate population size.^16^

The WHO SAT was used to comprehensively evaluate the hospital’s infrastructure, human resources, interventions, equipment and supplies.^13^ The WHO SAT consists of 176 items, each scored on a scale of 0 to 3 (0 – unavailable, 1 – inadequate, 2 – limited, 3 – adequate). The WHO SAT data were collected at the conclusion of the study period in December 2022. As this was a retrospective study, accurate baseline data from 2016 were not available. These findings therefore reflect the facility’s capacity at the end of the study period.

Data were checked, cleaned, aggregated and analysed using Microsoft Excel. A statistical process control (SPC) analysis of case volume was conducted and is graphically represented. Shifting means adjusted after eight successive points on the same side of the mean, denoting the central tendency. Upper and lower control limits were set at three standard deviations. This analysis technique is used widely in quality improvement projects and allows for changes in outcomes to be correlated with sentinel events which may affect outputs. The Pearson correlation coefficient (*R*) was used to assess the strength of relationships of normally distributed data.

A variable, ‘staff years’, was created to depict the relative contribution of each cadre of staff to the surgical service. It was calculated as a product of the cadre of each staff member and the number of years each staff member worked in that cadre over the study period.

Data were stored in a password-protected Google Drive folder, with access granted to a limited number of investigators.

### Ethical considerations

This study received approval from the University of Cape Town’s Human Research Ethics Committee (reference HREC 158/2023), as well as from the Eastern Cape Health Research Committee and the National Health Research Database (EC_202307_011). The hospital chief executive officer granted authorisation for data collection. Informed consent was waived as this was a retrospective review of hospital records; it was anonymised, with no direct patient contact or risk to participants.

## Results

### Surgical infrastructure

The hospital’s infrastructure was analysed using the WHO SAT.^13^ All five of the WHO SAT essential infrastructure items are usually available. These include electricity, running water, internet, oxygen and a 24-h emergency unit. However, the water supply depends on electricity, and the supply of both these essential utilities is not always reliable. All pharmacy items deemed essential by the SAT are available. Imaging modalities include X-ray (limited to office hours) and ultrasound (always available). Typically, four units of emergency blood are kept on-site, and basic laboratory services are provided. A blood bank and extended laboratory services are available at the referral centre only. The hospital meets all WHO SAT-specified operating room equipment and supplies requirements.

Surgical interventions are performed in three distinct areas: the main theatre – a dedicated environment for surgery only; the minor procedures room – an area within the emergency unit where complex resuscitations and minor procedures are performed; and the outpatient department rooms – consulting rooms where short, limited procedures are performed. Further details of the surgical facilities are provided in Table 1.

**TABLE 1:** Facility characteristics for Madwaleni District Hospital’s surgical service areas.

Operational characteristic	Main theatre	Minor procedures room	Outpatient department
Mode of anaesthesia provided	General, regional, local, sedation	Regional, local, sedation	Local
Surgical procedure examples	All major surgical procedures (caesarean sections, skin grafts, uterine evacuations, etc.)	Abscess drainage, tendon repairs, washouts, reductions, etc.	Minor excision biopsies, wound care, large loop excision of the transformation zone, etc.
Usual staffing per case	Non-specialist surgical provider, non-specialist anaesthetic provider, dedicated scrub nurse, dedicated floor nurse(s), midwife for caesarean sections	Non-specialist surgical provider, non-specialist anaesthetic provider as required and ad hoc nursing support only	Non-specialist surgical provider only
Dedicated central sterile supply department	Yes	No	No
Operational hours	24 h	24 h	24 h

### Surgical service delivery

#### Patient demographics

Of the surgeries conducted in the main theatre at the facility, 97% were performed on female patients. The median patient age was 25 years (range 2–89), with 10% of patients being minors (18 years old or younger).

#### Surgical volume

**FIGURE 1:**
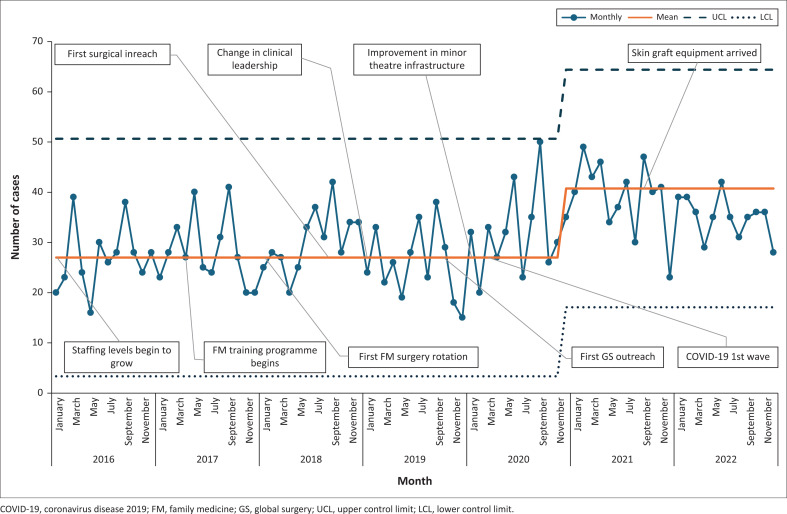
Statistical process control analysis of theatre volume in cases per month.

The trends in the volume of surgical procedures are shown in Table 2. A total of 2616 individual surgical cases were performed over the 7-year period. The volume of cases has increased over time, as visualised in the SPC analysis (Figure 1). The SPC analysis showed a significant upward shift in the mean monthly surgical volume, with the process mean shifting from an initial 27 operations to a new, sustained mean of 41 operations (as shown in Figure 1). Although multiple favourable local factors were present over the study period (such as intentional acquisition of surgical skills and equipment), the inflection points in case volume are not immediately preceded by any specific intervention or event. The caseload was predominantly comprised of CSs but also included 180 uterine evacuations, 124 perineal repairs following birth trauma and 28 skin grafts.

**TABLE 2:** Surgical procedure volume and scope at a Madwaleni District Hospital (2016–2022).

Surgical procedure	2016	2017	2018	2019	2020	2021	2022	Total	
	*n*	*n*	*n*	*n*	*n*	*n*	*n*	*n*	%
**General surgery**	**7**	**2**	**3**	**1**	**3**	**12**	**15**	**43**	**2**
Skin graft§	*1*	*1*	*1*	*0*	*1*	*10*	*14*	*28*	*1*
Debridement	*0*	*1*	*2*	*1*	*2*	*0*	*1*	*7*	*0*
Skin biopsy¶	3	0	0	0	0	1	0	4	0
Abscess incision and drainage‡¶	2	0	0	0	0	1	0	3	0
Lymph node biopsy¶	1	0	0	0	0	0	0	1	0
**Obstetrics and gynaecology**	**306**	**326**	**345**	**306**	**379**	**455**	**400**	**2517**	**96**
Caesarean section§	254	281	295	271	326	380	337	2144	82
Evacuation of retained products of conception§	32	21	21	20	17	42	27	180	7
Perineal repair	16	17	17	12	19	22	21	124	5
Female sterilisation§	0	2	10	1	3	0	1	17	1
Removal of retained intrauterine contraceptive device	0	0	0	2	7	2	5	16	1
Manual removal of placenta	2	2	2	0	3	1	2	12	0
Laparotomy for ectopic pregnancy§	0	3	0	0	1	1	2	7	0
Repair of cervical tear	2	0	0	0	0	3	1	6	0
Laparotomy for postpartum haemorrhage	0	0	0	0	2	1	2	5	0
Examination under anaesthesia	0	0	0	0	1	3	0	4	0
Marsupialisation of Bartholin’s cyst¶	0	0	0	0	0	0	2	2	0
**Orthopaedics**	**8**	**8**	**14**	**1**	**3**	**1**	**0**	**35**	**1**
Digit amputation¶	3	4	11	1	1	0	0	20	1
Lower limb amputation§	2	2	0	0	1	0	0	5	0
Tendon repair¶	0	2	2	0	0	1	0	5	0
Reduction of long bone fracture§¶	2	0	0	0	1	0	0	3	0
Irrigation and debridement of open fracture§¶	1	0	1	0	0	0	0	2	0
**Urology**	**3**	**3**	**2**	**2**	**1**	**4**	**6**	**21**	**1**
Hydrocelectomy§	0	0	2	1	1	3	6	13	0
Prostate biopsy	3	3	0	0	0	0	0	6	0
Male circumcision§¶	0	0	0	1	0	0	0	1	0
Orchidectomy	0	0	0	0	0	1	0	1	0
**Total surgical procedures, *n***	**324**	**339**	**364**	**310**	**386**	**472**	**421**	**2616**	-
**Total different types of procedures, *n***	**14**	**12**	**11**	**9**	**15**	**15**	**13**	**25**	-
Procedures per 100 000 population per year*, *n*	**331**	**349**	**378**	**324**	**407**	**502**	**452**	**391†**	-

* , Population size retrospectively modelled on recent census data.^16^

† , Mean value over the study period.

‡ , Denotes DCP3 primary health centre-level procedure.

§ , Denotes DCP3 district hospital-level procedure.

¶ , Denotes procedures performed in the operating theatre at the start of the study period, but shifted to the minor procedures room/outpatient department during the study period.

#### Surgical scope

Table 2 summarises the scope of surgical cases performed over the 7 years. A total of 25 different types of procedures were conducted. There were 14 different procedures performed in the first year, with an additional 11 different procedures performed over the remainder of the study period.

### Surgical workforce

**FIGURE 2:**
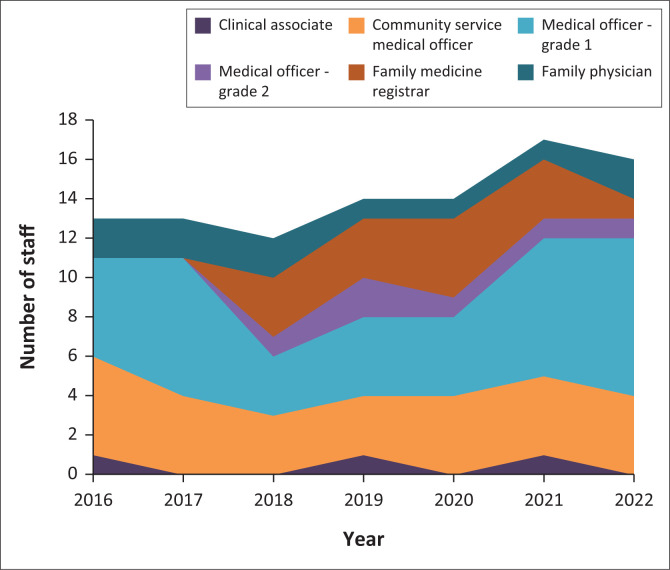
Surgical providers by cadre per year.

Figure 2 illustrates the composition of staffing over the study period. There was a statistically significant correlation between the number of cases performed and the total number of doctors employed (*r* = 0.86, *p* = 0.01). Whilst the number of junior staff (clinical associates, community service medical officers and grade one medical officers) remained relatively static, the number of senior staff (grade two medical officers, family medicine registrars and family physicians) increased. This qualitative change in staff coincided with the introduction of the family medicine training programme.

Table 3 shows the surgical volume per cadre per year, highlighting the relative contribution of different staff cadres to the surgical service. Family medicine registrars and family physicians conducted the most surgeries per clinician per year. Family medicine registrars provided the broadest range of surgical procedures, averaging nine different procedures per clinician per year.

**TABLE 3:** Workload per professional cadre.

Cadre of staff	Staff years*		Cases performed		Mean cases per clinician per year	Mean surgical repertoire per year	% Non-CS
	*n*	%	*n*	%	*n*	*n*	
Clinical associate	12	11	4	0	0.3	1	n/a†
Community service medical officer	27	25	388	15	14	4	15%
Medical officer – grade one	38	35	1037	40	27	7	17%
Medical officer – grade two	6	6	83	3	14	2	10%
Family medicine registrar	14	13	657	25	47	9	19%
Family physician	11	10	413	16	38	7	23%
Cadre not assigned‡	-	-	34	1	-	-	38%

**Total**	**108**	**100**	**2616**	**100**	**24**	**13**	**18%**

Note: Professional Cadres: Clinical associate – mid-level, non-physician, healthcare worker; Community service medical officer – medical doctor completed 2 years internship post-qualification; Medical officer – grade one: medical doctor with 0–5 years’ experience post-community service; Medical officer – grade two: medical doctor with 6–10 years’ experience post-community service; Family medicine registrar: family physician in training; Family physician: specialist, fellow of the college of family physicians, South Africa.

CS, caesarean sections.

* , Staff years – number of clinicians of this cadre multiplied by number of years they were present during the study period.

† , Not accredited to perform caesarean sections.

‡ , Surgical provider not recorded in theatre logbook.

### Theatre usage

An average of 1.02 cases per day was recorded over the study period. The operating theatre, available 24 h per day, was utilised for only 3% of the total available time. The median operative time for CS was 43 min, whilst the median operative time for all other cases was 31 min. Only six of the 24 non-CS procedures exhibited a longer median operating time than CSs. These procedures included wound debridement, skin grafts, laparotomy for ectopic pregnancies, orchidectomy, tendon repair and lower limb amputations.

## Discussion

Our study presents a detailed analysis of the surgical outputs of a rural DH in South Africa, showcasing the expanding surgical scope provided by a team of generalist practitioners. Between 2016 and 2022, the surgical service expanded in both the volume of cases performed and surgical scope. Whilst CSs predominate (82% of surgical volume), a wide range of surgical care has been provided to patients aged 2–89 years. These procedures account for eight of the 28 procedures in the World Bank’s Disease Control Priority 3 (DCP3) DH surgical basket of care. Furthermore, the WHO SAT analysis identified another five DCP3 procedures that were performed at the facility outside of the main theatre.^3^ The growth in surgical services is associated with an increase in total staff numbers, with participants in the family medicine specialisation programme making a notably large contribution to the service. The SPC analysis shows that no single intervention correlated directly with the increase in surgical capacity.

### Capacity for change

Caesarean sections dominated the theatre output at this facility, mirroring trends seen across South Africa and sub-Saharan Africa, where obstetric surgery typically accounts for 26% – 82% of procedures.^5,18,19,20^ This predominance is significant because the ability to perform CSs serves as a bellwether for broader surgical and anaesthetic competence.^12^ The presence of these essential skills, coupled with the observed low theatre utilisation rate, indicates likely substantial latent capacity to expand the service. Given that 58% of South African DHs perform CSs^21^ yet reportedly maintain low theatre utilisation rates,^22^ this unutilised capacity for expansion is likely a feature of other DHs.

Despite the expansion of the surgical service at this facility, the overwhelming predominance of obstetric and gynaecological procedures (96% of total volume) highlights a critical deficiency in the provision of general surgical, orthopaedic and trauma care. With only 99 non-obstetric and gynaecology procedures performed over 7 years, and in the absence of other district-level hospitals in the immediate catchment area, it is evident that the non-obstetric surgical burden is not being met locally. This implies that patients are either transferred to distant tertiary referral centres – placing strain on higher levels of care – or face barriers that leave their health needs unmet. This disparity identifies a major gap in local responsiveness to the community’s broader burden of disease.

We have shown that at this DH, there is a significant positive correlation between the number of surgical providers and the volume of surgeries performed. This highlights the critical role of workforce capacity in meeting surgical demands. Exploring the role of clinical associates in expanding surgical services at DHs in South Africa could be an important step in addressing limited staffing capacities in these settings. Although CS competency often predicts broader surgical capability,^12^ the reverse is not necessarily true; competency in general surgical procedures does not require CS accreditation. Thus, clinical associates remain a vital resource for addressing the non-obstetric surgical gap. By taking on minor and intermediate procedures such as debridements and evacuations, they can expand the hospital’s general surgical capacity while offloading medical officers to manage the high burden of obstetric emergencies. Similar task-shifting models in other LMICs have successfully enabled mid-level healthcare workers, such as clinical associates, to provide safe and effective surgical care, improving access and outcomes in resource-constrained environments.^20,23^

### Drivers of change

#### Staff

Staff numbers are widely acknowledged as critical to surgical service delivery.^2^ Several studies suggest an important correlation between increased staff numbers and increased surgical volume.^24,25,26,27^ However, in sub-Saharan Africa, an increase in staff does not always result in a proportional increase in surgical volume at DHs.^28^ At our hospital, more experienced staff and those enrolled in a training programme (family medicine registrars) made a notably large contribution to the surgical output.

In 2017, the health sciences faculty of the nearest university instituted a decentralised family medicine specialisation programme, changing the training model by facilitating training at DHs rather than urban university hospitals. These specialists in training are tied to their DHs for 4 years, creating continuity and building experience. They are incentivised to grow skill sets through logbooks and competency requirements, and the curriculum deliberately targets systems’ improvement skill sets such as leadership and quality improvement methodology. In the study hospital, this cadre of staff members, in combination with the scaffolding and incentives for growth in their programme, have been influential in expanding surgical services.

Our findings suggest that the introduction of this family medicine training programme was a primary driver of the observed expansion in surgical volume and scope. Whilst we cannot quantify a specific lag time or prove direct skills transfer without further qualitative research, the high individual productivity of these registrars (performing 25% of cases) suggests their contribution was critical to the service.

#### Accessibility of the next procedure

There have been multiple local and international attempts to define a basket of surgical care appropriate for DHs, but because they represent a heterogeneous group of healthcare facilities with no standardised definition in terms of size or services offered, consensus on a set of surgical norms that is practical and implementable has proven elusive and sometimes contradictory.^3,9,11^ An alternative approach to designing and rolling out a ‘basket of care’ is to realise incremental growth through the next single, achievable addition to the surgical repertoire.^29^

Lessons learned and capacity gained with the introduction of a novel procedure can create a platform for systems towards the provision of ever more complex care. Choosing the next procedure to introduce requires balancing the capacity of the staff and facility against the impact of a particular procedure on patient-specific outcomes and the local disease burden. Contextual factors are important and may make specific procedures attractive targets despite their complexity, as occurred in another rural DH where a preponderance of hip joint arthritis drove the introduction of hip arthroplasty surgery.^30^ At Madwaleni Hospital, the local team targeted accessible procedures such as tendon repairs, hydrocelectomies and split-thickness skin grafts where local demand was significant, barriers to performing the cases were low and access to the procedures at referral centres was limited. Surgical, anaesthesia and nursing competence gained through performing these procedures has made the team more confident to perform higher complexity procedures. Locally, concerns remain around the total volume of certain procedures performed because they do not yet meet thresholds for the high volumes of repetition associated with improved patient outcomes for general practitioners over specialists.^31^

Other strategies for facilitating surgical repertoire expansion include supervised exposure to cases through specialist outreach to the rural facility or visits by DH staff to urban referral centres, retention of senior doctors in rural DHs and short courses in surgery.^32,33^ However, aiming to improve a system to provide an entire basket of care at a single DH may not be as achievable as providing a surgical basket of care within the wider referral system of a health district. One locally discussed proposal is to create DH centres of excellence for certain procedures (i.e. one DH specialises in burn care whilst another performs hernia repair). This would generate referral pathways between DHs that offload tertiary referral hospitals and create the higher volumes required for improved competence.

### Measuring change

#### Staff

District hospitals are essential vehicles for improving access to surgery.^2,3,34^ In LMICs, these facilities are often staffed by non-specialist surgical providers.^2,5,28,35^ Current benchmarks for measuring surgical provider density are focused on the density of surgical specialists,^2^ but the density of surgically skilled generalists may be a more important predictor of access to surgical care at DHs. Data regarding the number of non-specialist surgical providers are not readily available in South Africa.

#### Procedures

National and international bodies have called for countries to collect and compile data on the number and types of surgical procedures conducted to inform policy and improvement initiatives. South Africa lacks a systematic method for gathering this information.^25,34^ Data collected through the National Indicator Data Set database only include surgical data for cataracts, CSs, sterilisations (male and female), male circumcisions and dental extractions.^36^ The data for this study were extracted from a hardcopy surgical registry based in the operating theatre and completed by nurses. These registries are mandated and present in all theatres in DHs and thus offer an untapped data source. However, accessing this data is laborious, and data on the unmet surgical needs are excluded.

### Limitations

This study, whilst adding valuable longitudinal data to the sparse literature on DH surgical services, has several limitations inherent to its retrospective design and data source. Firstly, the quantitative analysis relied exclusively on the main operating theatre register. Consequently, surgical activity performed in other clinical areas – such as the casualty unit and outpatient consultation rooms – was not captured. This includes procedures such as orthopaedic reductions, tendon repairs, open fracture washouts and biopsies. Notably, some of these procedures were performed in the main theatre at the start of the study period but were subsequently shifted to these alternative settings to improve efficiency. Their disappearance from the register therefore represents a change in workflow rather than a cessation of service, leading to an underestimation of the total facility volume and an apparent reduction in the recorded scope.

Secondly, the data extraction was limited to the ‘primary procedure’ recorded for each case. Secondary procedures performed concurrently (such as bilateral tubal ligations during CSs) were not quantified. This further contributes to the under-reporting of the total surgical output.

Thirdly, the exclusive use of the main theatre register resulted in a marked under-representation of trauma-related procedures, which are frequently managed in the casualty unit or referred directly to tertiary care. As a result, this study does not capture the surgical burden related to trauma in this region.

Finally, we did not record quality of care measures, patient outcomes, or complication rates. As a result, our understanding of the effect of the expansion of this DH surgical service is limited to volume and scope, rather than quality.

Without robust mechanisms to describe the total community demand for surgery or the rates of referral to tertiary centres, we cannot fully quantify the unmet need. However, the low absolute volume of non-obstetric surgery suggests a significant reliance on referral networks for general surgical and trauma care.

## Conclusion

District hospitals are uniquely positioned to play a critical role in the delivery of EESC in LMICs. Their geographical proximity to communities, the prevalence of functioning operating theatres and the availability of appropriately skilled staff make them vital components of the surgical ecosystem.

In South Africa, however, there is a lack of quality data to guide the expansion of surgical services at DHs. Key gaps include an understanding of the determinants of successful surgical service expansion and its impact on both communities and other clinical services in the already overburdened DHs and referral facilities.

Our findings suggest that two key interventions – the introduction of a decentralised family medicine training programme and the availability of an adequate and experienced workforce – may have been key interventions for increasing surgical volume and scope at this DH. With proper support, and by aligning comprehensive service delivery with decentralised training, DHs have the potential to provide scalable, context-driven solutions to bridge the significant gap between met and unmet surgical needs in resource-limited settings.
